# Craniofacial Bone Tissue Engineering: Current Approaches and Potential Therapy

**DOI:** 10.3390/cells10112993

**Published:** 2021-11-03

**Authors:** Arbi Aghali

**Affiliations:** 1 Department of Physiology and Biomedical Engineering, Mayo Clinic, Rochester, MN 55905, USA; aghali.arbi@mayo.edu; 2 Weldon School of Biomedical Engineering, Purdue University, West Lafayette, IN 47908, USA

**Keywords:** MSCs, MKs, growth factors, BMP-2, TPO, biomaterials, craniofacial bone regeneration, tissue engineering

## Abstract

Craniofacial bone defects can result from various disorders, including congenital malformations, tumor resection, infection, severe trauma, and accidents. Successfully regenerating cranial defects is an integral step to restore craniofacial function. However, challenges managing and controlling new bone tissue formation remain. Current advances in tissue engineering and regenerative medicine use innovative techniques to address these challenges. The use of biomaterials, stromal cells, and growth factors have demonstrated promising outcomes in vitro and in vivo. Natural and synthetic bone grafts combined with Mesenchymal Stromal Cells (MSCs) and growth factors have shown encouraging results in regenerating critical-size cranial defects. One of prevalent growth factors is Bone Morphogenetic Protein-2 (BMP-2). BMP-2 is defined as a gold standard growth factor that enhances new bone formation in vitro and in vivo. Recently, emerging evidence suggested that Megakaryocytes (MKs), induced by Thrombopoietin (TPO), show an increase in osteoblast proliferation in vitro and bone mass in vivo. Furthermore, a co-culture study shows mature MKs enhance MSC survival rate while maintaining their phenotype. Therefore, MKs can provide an insight as a potential therapy offering a safe and effective approach to regenerating critical-size cranial defects.

## 1. Introduction

Large cranial defects can result from a variety of conditions, including congenital defects [1,2,3,4], tumor resection [3,5,6], infection [5,6], and severe trauma [3,4,6,7]. Critical-size cranial defects can leave a large area of the brain unprotected with a significant cosmetic deformity [4,7]. In addition, cranial defects cause a myriad of symptoms that affect the quality of people’s lives, including headache [8,9,10], dizziness [11,12,13], irritability [11,12,13,14], depression [13,15,16,17,18], anxiety [13,16,17,19], intolerance to noise and vibrations [12,13,17,18], and inability to concentrate [12,13,17]. Furthermore, a progressive soft tissue deformity has been observed in patients [19,20], causing neurological deficits [19].

Bone tissue engineering is a promising approach that utilizes MSCs, growth factors, and scaffold biomaterials to induce new bone tissue formation [21,22,23,24]. Bone-grafting methods, such as autograft, allograft, and xenograft, have shown positive results [25,26,27,28,29,30]. However, limitations exist, including donor site morbidity and inconsistent outcomes [21,28,31,32,33]. On the other hand, natural and synthetic biomaterials have shown optimistic outcomes for reconstructive applications [23,34,35,36,37]. For instance, natural biodegradable polymers such as collagen and fibrin are investigated for bone tissue engineering applications to overcome restrictions caused by synthetic/non-degradable biopolymers [13,23,34,35,36,37]. In addition, synthetic biomaterials such as Polymethyl Methacrylate (PMMA) and Calcium Phosphate Cement (CPC) show reconstructive integration and ability to match irregular patient defects [38,39,40].

**Figure 1:**
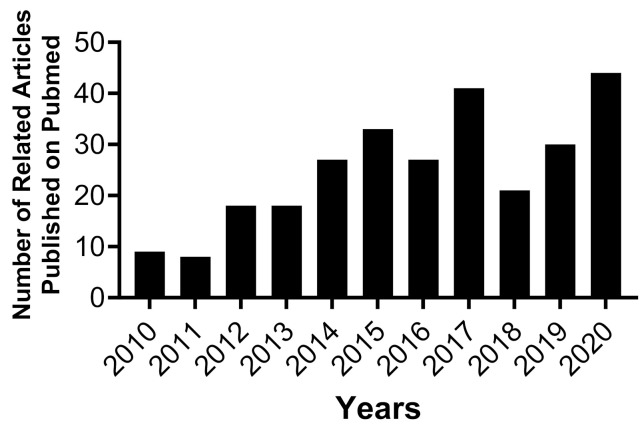
The number of articles published in the United States National Library of Medicine (PubMed) between 2010 and 2020. The keywords used in search engine database are “Craniofacial Bone Tissue Engineering + Mesenchymal Stromal Cells”. Data are collected between 2010 and 2020. Data show a steady increase in the numbers of published articles between 2010 and 2020. Numbers of papers related to the keywords published in 2010 are nine, while forty-four papers were published in 2020. Increasing number of published articles indicates an interest in investigating mesenchymal stromal cells for craniofacial bone regeneration.

Equally important, MSCs have been identified as a key player in bone maintenance and repair [3,24,31,41,42,43,44,45,46,47,48,49]. Autologous MSCs using Bone Marrow Mesenchymal Stromal Cells (BMSCs) and Dental Pulp Mesenchymal Stromal Cells (DPSCs) promote healing and recovery in various animal models and patients with significant bone defects [3,12,33,50]. BMSCs and DPSCs are being investigated for craniofacial bone tissue engineering applications for several reasons. For instance, there are site-specific differences between long bone and cranial bone tissues concerning bone repair and remodeling mechanisms [51]. Furthermore, BMSCs and DPSCs originate from two different germ layers during embryogenesis [50,52,53]. Additionally, Proliferation and differentiation capacity of BMSCs and DPSCs are distinct [54]. Studies have revealed that DPSCs show higher proliferation and differentiation capacity than BMSCs harvested from the same species [26,55,56]. As a result, a steady increase in the number of published articles shows an interest in further understanding and utilizing MSCs for craniofacial bone tissue engineering purposes in the past decade [55,56,57,58,59,60], Figure 1.

Adding growth factors to scaffold biomaterials enhances MSC differentiation and ultimately promotes a new bone formation. Several growth factors are being investigated to facilitate MSC differentiation into a desired cell type [61,62]. Specifically, Bone Morphogenetic Proteins (BMPs), a group of multifunctional growth factors that belong to the Transforming Growth Factor-Beta (TGF-β), play an essential role in inducing new bone tissues [63,64,65]. Various BMP family members such as BMP-2, BMP-6, and BMP-7 contribute to boosting a new bone tissue formation [63,64,65,66]. The impact of BMP family on MSC differentiation has been investigated by researchers in the field and shows possibility [12,45,61,67,68,69,70,71,72,73,74,75,76], the most prevalent is BMP-2 [61,62]. BMP-2 is a potent osteoinductive growth factor that plays a vital role in bone formation and repair [61,77,78,79]. Therefore, BMP-2 is used as a treatment when delivered into a defect site via, for example, collagen sponge [77,78,79]. This approach shows promising results regenerating a critical-size long bone defect and maxillofacial osseous fractures [77,78,79].

While BMP-2 has demonstrated positive results in inducing a new bone tissue formation, BMP-2 can also cause excessive ectopic bone formation [80,81,82,83,84,85,86,87], causing cosmetic deformities [80]. Furthermore, BMP-2 induces treated cells to release inflammatory risk factors such as IL-6 [87,88,89,90,91], IL-10 [89], and TNF-α [89,90,91]. Elevated levels in these inflammatory factors are associated with cellular senescence and defined as the hallmarks of aging cells [92,93,94,95,96,97]. Furthermore, other studies have reported controversial results of BMP-2, connecting BMP-2 to facilitate carcinogenicity production [98,99,100,101,102,103].

**Figure 2:**
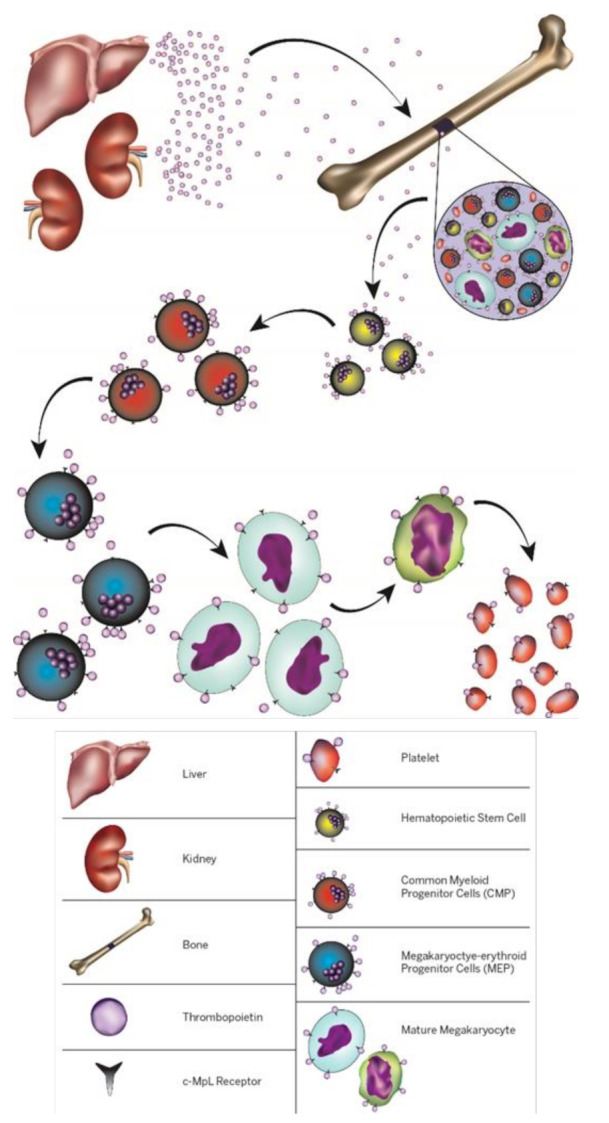
A schematic of MKs production. MKs are hematopoietic cells responsible for platelet production. Journey of megakaryopoiesis starts when TPO targets Mpl receptor expressed on hematopoietic stem cells. Next, hematopoietic stem cells differentiate through a hierarchical series of colony-forming units (megakaryocytes (CFU-MK)). Ultimately, MKs undergo a maturation process producing platelets.

Another growth and development factor is TPO. TPO, a primary regulator of MKs [104,105,106,107], has been an active research area by multiple research groups. Mpl receptor of TPO is expressed on several cell types, such as Hematopoietic Stem Cells (HSCs) and MKs [107,108,109]. TPO is a protein produced by liver and kidney. TPO induce HSCs to differentiate into mature MKs which are ultimately producing platelets, Figure 2 [104,105,106,107,110,111]. Several studies demonstrate that MKs play a key role in osteoblast proliferation [108,112,113,114,115] and osteoclast formation [114,115,116,117,118,119]. Osteoblasts and osteoclasts are essential for bone remodeling and eliminating necrotic tissue in early bone repair [120,121].

Moreover, the role of MKs regenerating a critical-size bone defect is an ongoing and active area of research, utilizing in vitro cell culture system [56,112,116,122,123,124]. However, whether or not MKs can facilitate MSCs proliferation and differentiation to regenerate cranial bone defects is a question yet to be addressed. Particularly, the effects of increasing MK count to enhance viability and differentiation of BMSCs or DPSCs in vivo for cranial bone tissue engineering purposes has not been determined [125].

However, over productions of MKs for craniofacial regeneration purposes can have a downside effect. Studies have shown that increasing MKs, and ultimately platelets can be a potential risk causing bone marrow fibrosis [122,126]. Furthermore, a co-culture study of MKs with MSCs has reported that MKs inhibit MSC differentiation into osteoblast lineage cells by suppressing expression of ALP activity and calcium deposition [125].

While progress has been made in tissue engineering and regenerative medicine field, several challenges remain. For instance, although BMP-2 has demonstrated promising results in regenerating large cranial bone defects, better outcomes are desired. Furthermore, the need for an additional growth factor rises from the excessive effects of BMP-2 and low survival rate of MSCs post-implantation. Herein, we discuss current craniofacial bone tissue engineering approaches. This review aims to identify advantages and challenges of current and proposed solutions in craniofacial bone tissue engineering field. Specifically, we will review methods currently used to restore cranial bone defects, such as natural and synthetic bone graft substitutes, MSCs (BMSCs and DPSCs), and current growth factors commonly used for cranial bone regeneration. Next, we will present a potential therapy; a discussion on rationale of inducing MKs for therapeutic applications to facilitate craniofacial bone regeneration.

## 2. Craniofacial Bone Tissue Engineering: Current Approaches and Challenges

Craniofacial bone tissue engineering utilizes a synergistic combination of MSCs, growth factors, and scaffold biomaterials [21,22,23,24]. The combined model can promote new bone formation to regenerate a critical-size cranial defect [23,24,26]. However, alternative methods using bone grafts such as alveolar ridge [127] and maxillary sinus floor [123] have shown promising results. Clinical studies have demonstrated effective use of allograft scaffolds harvested from calvarial bone and mandibular condyle of temporomandibular joint [124,128]. Several steps are necessary to repair critical-size bone defects successfully. The desired implanted scaffold should meet the following criteria: (1) Deliver and/or recruit naïve cells into the defect site [23,129,130,131,132]; (2) incorporate growth factors into biomaterial scaffolds [133,134,135]; (3) allow vascularization and new bone tissue formation [136,137,138]; (4) facilitate exchange of nutrient and oxygen in vivo [133,136,137,139]; (5) stand a high structural load-bearing capacity [135,136,137,140]; and (6) ability to support and balance new bone tissue formation and scaffold degradation [133,134,135,136,140,141]. These requirements appear to be critical for effective craniofacial bone regeneration [23,135,142,143].

## 3. Biomaterials for Craniofacial Bone Regeneration

### 3.1. Autologous Bone Graft

Bone grafting is a surgical procedure that aims to replace missing bone using tissue harvested from patient’s skeleton (autograft) [32,134,135,144], donor (allograft) [32,134,138,145,146] or different species (xenograft) [32,34,147,148,149]. However, bone autografting has showed positive outcomes regenerating cranial bone defects [140,141]. An autograft is a procedure using bone tissue as a substitute harvested from patient’s different anatomical location and transplanted into the defect site [32,149,150,151]. In other words, bone graft is harvested from one side of patient’s skeleton into cranial defect site [147,150,151,152]. The graft substitute of autologous bone can be harvested from a variety of locations. Common locations are tibia and iliac crest [142,143,147,153,154]. Autograft is a safe surgical procedure moving tissue from one side to another [30,142,143,153]. Using patient’s bone tissue minimizes risk of immune system reactions and transferring pathogens from one source to another [32,151,153,155]. In addition, autografts have several advantages: Enabling osteogenesis [25,145,152,156,157], osteoinduction [25,145,148,152,156,157], and osteoconduction [149,152,156,157]. All of which are essential to promote new bone formation [152,157].

On the other hand, bone autograft may require rearrangement for two surgical procedures [142,153]. As a result, patients face extra pain and possible blood loss due to the required two surgeries [157,158,159,160]. Moreover, the autograft procedure may require an extended hospitalization to a mandatory care service [153,155,161]. Consequently, this may yield a higher cost for patients [162]. Other disadvantages are increasing pain, scar at donor site, and extra damages to surrounding healthy tissues such as nerve, bone, and blood vessels [142,143,147,149,150,153]. Moreover, patients with pre-existing conditions such as diabetes may not be eligible for bone autografting [159,163,164].

Another factor to consider is patient’s age and health condition [151]. The age of patient can be disadvantage using autograft reconstruction [151]. For instance, harvesting bone graft for children can cause complications and pain [151]. Moreover, autograft substitute may not be an optimal decision for children unless their cranium is fully established and can better withstand the impact of significant surgery [142,158]. Therefore, children might be less likely suitable candidates for bone autograft procedure [151,158,159,165]. Similarly, aging adults with medical conditions such as neuromuscular scoliosis have shown insufficient bone healing [138,163]. Table 1 summarizes the advantages and disadvantages of bone autograft.

### 3.2. Allogeneic Bone Graft

Although autograft bone is a desired approach to regenerate critical-size cranial bone defect [32,148,153,174], allograft has been an attractive and alternative method [32,35,148,153,174]. Allograft bone tissue is transplanted to the patient (recipient) from a donor of the same species (human) [23,32,147,153,168]. In the United States, the number of bone allogenic grafts surgery is steadily increasing [33,174]. In the last decades, the number of surgeries involving allogeneic grafting tissue increased more than 15 times [147,166,168,175]. The main advantages of bone allografts are to provide structural support [32,166,176], decrease surgical time [32,149,176], and promote cranial healing [149,175,176]. The three most common bone allograft types are cortical, cancellous, and hybrid bone tissue (cortical and cancellous bone tissue) [23,157,171]. Each one of harvested bone substitute has advantages. For instance, the cancellous bone is a desired bone graft for cranial reconstruction [161,166,167,177]. This is due to bone’s elasticity and sufficient pore size that allow for cell infiltration and nutrient and gas exchange [23,172,178,179,180]. Furthermore, mechanical and structural properties of cancellous bone allow for new bone and blood vessel formation [177,178,181]. On the other hand, cortical bone is a less favorable bone graft due to its low osteoconductivity and resorption rate [172,178,180].

Nevertheless, patients who receive allogeneic transplant from a deceased donor pose some risks [33,168]. Although bone allograft remains safe (the case of viral transmission is low), concerns regarding the safety of allografts remain [168,172]. On a rare occasion, unexpected transmission of pathogen such as Human Immunodeficiency Virus (HIV) from donor to the recipient can occur, despite donor screening to rule out possibility of donor infection [147,168,182]. In addition, potential risk of unwanted immune response, transplant rejection, and allergy may present challenges [35,182,183].

Various methods to prepare allograft bone for transplant have been used. One of the common practices is to debride donated tissue and sterilize it, followed by lyophilizing tissue to destroy any remaining living cells in bone tissue [155,157]. Although this method has shown constructive outcomes, an optimal procedure to clean, sterilize, and remove cellular and biological constituents from bone substitutes is desired [33,178]. For instance, when bone tissue undergoes lengthy cleaning, sterilization, and decellularization processes (such as lyophilization), a meaningful decrease in mechanical strength and structure of bone tissue occurs [168,170]. However, enhancing weight-bearing of allograft bone with a polymer composite is proposed and shows promising results [183]. Table 1 summarizes advantages and disadvantages of allografts.

### 3.3. Xenogeneic Bone Graft

A xenograft is a procedure of transplanted bone tissue harvested from different species to the patient. Bone tissue is prepared by physical or chemical processing and implanted in the patient (recipient) [166,170]. The most common sources of xenogeneic grafts are bovine and natural coral [149,170]. Similar to allogeneic bone grafts, xenogeneic bone graft serves as a structural load-bearing scaffold to facilitate new bone tissue growth and fill the vacant defect [29,184]. Unlike allograft, xenograft reduces the risk of transmitting human diseases caused by transmitted pathogen from the donor to recipient [31,172,185]. On the other hand, xenogeneic bone grafts present potential risks [173]. For instance, immunological barrier to xenotransplantation and potential of transmitting infectious diseases are a concern for tissue engineering and medical community [31,185,186,187]. Furthermore, the unique sterilization process, such as exposing harvested bone to a high temperature deteriorates mechanical and structural properties of the bone graft and reduces osteogenic and osteoinductive properties [149,157].

However, despite poor outcomes reported from xenogeneic bone grafts [149], xenotransplantation remains a standard and successful procedure in dental applications [148,188,189]. To overcome challenges presented by xenogeneic bone grafts, bone graft has been combined with growth factors [190,191] and, in other cases, with allogeneic or alloplastic bone substitute to serve as a hybrid scaffold [175,191,192]. This approach has shown promising outcomes by inducing new bone tissue [193,194,195]. Table 1 summarizes advantages and disadvantages of xenograft.

### 3.4. Alloplastic Bone Graft Substitute

An alloplastic bone substitute is a biocompatible material that is produced synthetically by physical or chemical processing. In recent years, alloplastic bone substitutes have gained more attention, mainly in craniofacial bone reconstruction [170,176]. While surgical procedure to repair cranial defects is known as cranioplasty [35,40,177], the term of alloplastic bone substitute is associated with synthetic biomaterials [160,166]. Alloplasty is a procedure that substitutes large missing bone with synthetic biomaterials to bridge the fracture [35,172,196]. Notable reasons that make alloplastic biomaterials desirable in cranial repair are unlimited availability, elimination of the need for donors, and minimize potential risk of pathogen transmissions [35,172,190]. Another advantage of alloplastic biomaterials is that patients may not require a second surgery [161,166].

Promising alloplastic bone substitute for craniofacial bone regeneration should be biocompatible and biodegradable [23,37,48,143,197], non-genotoxic [179,180,198,199], non-carcinogenesis [199,200], non-inflammatory [86,197,201], and bioresorbable [36,133,148,197]. Furthermore, alloplastic bone substitutes should have an appropriately interconnected porosity and a satisfactory porous size (approximately 200 to 300 µm) for permeability and guidance of new bone tissue formation [161,166]. Scaffold biomaterials that meet these criteria have been shown to enhance new bone regeneration and minimize potential reaction of immune system [133,148,172,186].

Several organic and inorganic biomaterials meet these requirements, among them are PMMA and CPC. PMMA is used as a biomaterial for craniofacial reconstruction [182,186,202]. PMMA is routinely used in cranioplasty due to its desired mechanical stability in vivo [40,203,204], and is widely available and affordable [185,186]. Furthermore, PMMA can be molded into shape to match irregular patient defects [38]. Although PMMA has several advantages that make it suitable biomaterial for cranial bone regeneration, other disadvantages are reported. For instance, PMMA is non-biodegradable polymer. However, it has been used for applications that require a permanent implant, such as dental applications [187,205]. Furthermore, PMMA may display insignificant integration with surrounding tissue, including bone tissues [187,205].

On the other hand, CPC is a promising biomaterial that has been studied to repair cranial bone defects [41,42,206]. The desirable prosperities of CPC include ease to shape and contour, as well as ability to enhance osteoconductivity [188,189]. These unique properties make CPC an attractive and alternative biomaterial for craniofacial bone regeneration. However, one of CPC disadvantages is brittleness. Reinforcing CPC with biopolymers to serve as a composite biomaterial is proposed [40,193,194,207]. Nevertheless, combination of alloplastic biomaterials with MSCs has shown encouraging results [61,64,67].

Another aspect affecting MSC differentiation into target cell lines is the interaction between cells and the surface of biomaterials. It has been established that scaffold topography of biomaterials influences MSC fate, including migration, differentiation, proliferative capacity, and adherence. Recently, two separate studies showed that 60.66 µm and 32.97 nm pore sizes of distinct nano- and microtopography of wet Spongostan are sufficient to facilitate osteogenic differentiation of human stromal cells in vitro [195], as well as to induce new bone formation in a critical-size calvarial rate in vivo [195,208].

## 4. Mesenchymal Stromal Cells: Successes and Challenges

Stem cell therapy has been an active research area to overcome challenges face tissue engineering and medical community [196,197,209,210]. Several studies have demonstrated a successful transition of stem cells to the patients. Research studies have enriched the hope that this regenerative approach may become a treatment for a wide range of critical-size craniofacial bone defects. However, researchers are exploring multiple stem cell types to regenerate critical-size craniofacial defects, including MSCs.

MSCs are undifferentiated cells with two unique properties: Self-renewal and differentiation into the desired cell type [201,211,212,213,214]. MSCs can facilitate cranial defects by differentiating into osteoblasts to repair damaged tissues and to restore craniofacial functions [56,60,215,216]. MSCs are harvested from multiple anatomical locations, such as bone marrow and dental pulp tissues [196]. MSCs can be found in multiple locations in skeleton tissues, including adipose, dental pulp, bone marrow, and periosteum [203,204,217,218]. However, growing evidence indicated that MSCs are not the only distinct and differentiated cells that can regenerate defective bone tissue. Other cell types can dedifferentiate and participate in tissue formation in some species. For example, Knopf et al. have shown that osteoblasts can dedifferentiate by downregulating bone markers, and upregulating bone progenitor markers to participate in forming blastema in zebrafish [219]. However, as research advances, emerging evidence shows a distinct type of MSCs in their capacity to repair bone defects. For instance, Mizuhashi et al. have demonstrated that stem cells in the periosteum of mice have higher bone regeneration capacity than BMSCs [204].

Furthermore, a controversy has been raised on MSC-based origins, particularly what type of MSCs can be more desired to regenerate a specific bone fracture? For example, what a desired MSC type (BMSCs or DPSCs) can regenerate craniofacial bone defects? This section will focus on two MSC types used in craniofacial bone tissue regeneration: Bone Marrow Mesenchymal Stromal Cells (BMSCs) and Dental Pulp Stromal Cells (DPSCs) [196]. BMSCs and DPSCs have unique properties that make them attractive candidates for craniofacial bone regeneration [220,221,222].

### 4.1. Bone Marrow Mesenchymal Stromal Cells (BMSCs)

BMSCs are adult multipotent stem cells derived from bone marrow tissue [47]. BMSCs are a promising cell source due to their self-renewal and multipotency by differentiating into different cell types, such as osteoblasts, chondrocytes, and adipocytes [125,206,209,223,224,225,226,227,228,229]. BMSCs are attractive MSCs that have high therapeutic potential. BMSCs can proliferate in vitro and can be used in clinical applications without losing their capacity [215,216,230,231]. BMSCs have demonstrated potential to regenerate cranial bone defects. A new bone formation is observed when BMSCs are harvested, culture-expanded, and implanted in calvarial bone defect of rabbit animal model [24,220,232,233]. This procedure demonstrates efficacy in repairing a cranial bone defect. Furthermore, promising results have been shown when autologous BMSCs are used. The transplant of autologous BMSCs is vital to avoid unwanted immune system responses [46,52,215,231,234].

**Figure 3:**
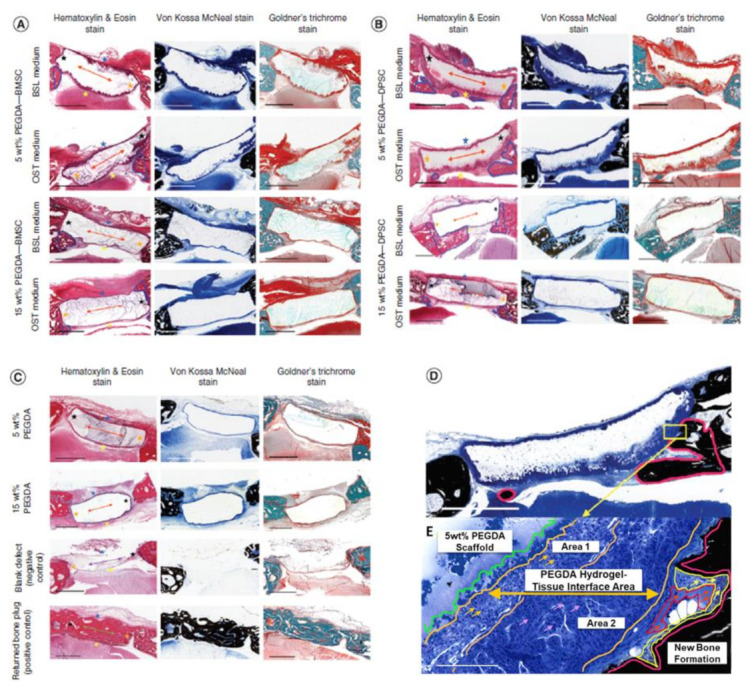
Histological cross-section views of retrieved specimens harvested from calvarial defect model of New Zealand White rabbits, 6 weeks post-implantation. Specimens were collected and stained with Hematoxylin and Eosin (H&E), Von-Kossa MacNeal’s, and Goldner’s Trichrome. (**A**) shows photoencapsulated-BMSCs in 5 or 15 wt% PEGDA hydrogels, cultured in basal (BSL) or osteogenic (OST) medium for 3 days prior to surgery. (**B**) shows photoencapsulated-DPSCs in 5 or 15 wt% PEGDA hydrogels, cultured in basal (BSL) or osteogenic (OST) medium for 3 days prior to surgery. (**C**) shows control groups: 5 or 15 wt% PEGDA hydrogels without MSCs, a blank defect (a negative control group), and a returned bone plug (a positive control group). The blue irregular lines in H&E stain (**A**,**B**) show new bone tissues. Orange two-sided-arrows in H&E stain (**A**–**C**) show remaining hydrogel scaffolds, while blue star shows dorsal. Yellow stars show ventral, black star shows medial, and orange star shows lateral. (**D**) shows photoencapsulaed-DPSCs in 5 wt% PEGDA hydrogel. Red irregular line illustrates a new bone tissue. (**E**) is amplification of selected area in (**D**). The green line on upper left side indicates remaining hydrogel scaffold area, while orange lines show the hydrogel-tissue interface area. Orange and pink one-sided arrows in sub-interface areas, one and two, show blue-stained nuclei. One-sided red arrows inside irregular red line areas show osteoid. Scale bar is 2 mm [24].

BMSCs act as reservoirs of reparative cells. They have been identified as key players in bone maintenance and repair [215,216,235]. Accordingly, there has been an increasing interest in using BMSCs for cranial bone regeneration. Recently, we have shown that photoencapsulated BMSCs in fast degrading thiol acrylate hydrogels promote new bone formation in rabbit calvarial defects, compared to negative control group, 6 weeks post-implantation [24,47], Figure 3. Other studies have shown similar conclusions using BMSCs to regenerate critical-size bone defects [50,236,237]. BMSCs have several advantages. Studies show encouraging outcomes in restoring critical-size cranial fracture utilizing animal models. However, one challenge facing research communities is maintaining BMSCs phenotype in tissue culture dish, particularly during proliferation and passaging in vitro. When BMSCs attach to the surface of tissue culture dish, they tend to activate and upregulate key bone markers [125,238,239,240].

Furthermore, BMSCs tend to age and lose their proliferation capacity with advanced passage numbers [215,241,242]. Advanced passage numbers of BMSCs show an increase in senescence markers, where BMSCs enter G_1_/S phase of cell cycle arrest [216,243]. Ridzuan et al. have reported rat BMSCs show a decline in cell growth at advanced passage number of four [216]. Moreover, senescence beta-galactosidase stain, an enzyme-based assay that identifies senescent cells in culture, is increased at passage number of five [216]. The authors concluded that advanced passage number of BMSCs meditated cellular senescence by limiting BMSCs growth [216]. Other studies have reached a similar conclusion demonstrating the impact of using prolonged and advanced passage number of MSCs on their potential use for regenerative or research purposes [197,221,243,244,245,246,247].

One of the limitations of BMSCs for bone tissue regeneration is low survival rate of BMSCs after transplantation [222,235,248,249]. The low survival rate of BMSCs post-transplantation is crucial for researchers and physicians [220,222,235,248]. The harsh native microenvironments such as inflammation and immune system response, mechanical leakage of BMSCs after injection, cell necrosis and apoptosis, and imbalance in radicals and antioxidants can lead to BMSCs loss [235,250]. Moreover, low survival rate of BMSCs can limit their self-renewal capacity due to lack of nutrients, ECM production, and oxygen [235]. Despite challenge, several techniques have been explored to overcome these obstacles—notably, more effective methods delivering BMSCs.

**Figure 4:**
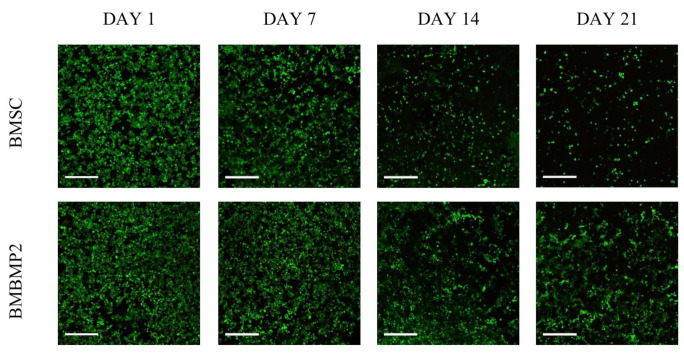
Immortalized mouse BMSCs photoencapsulated in visible light-cured thiol-acrylate hydrogels. Confocal microscopy images of photoencapsulated-BMSCs with or without BMP-2: photoencapsulated-BMSCs without BMP-2 (BMSC), and photoencapsulated-BMSCs with BMP-2 (BMBMP2). BMSCs were stained with a live/dead cell staining kit (green = live cells, red = dead cells). Images were taken on days 1, 7, 14, and 21. Greater BMBMP2 viability is observed at days 14 and 21 compared to BMSCs. The scale bar is 100 µm [47].

**Figure 5:**
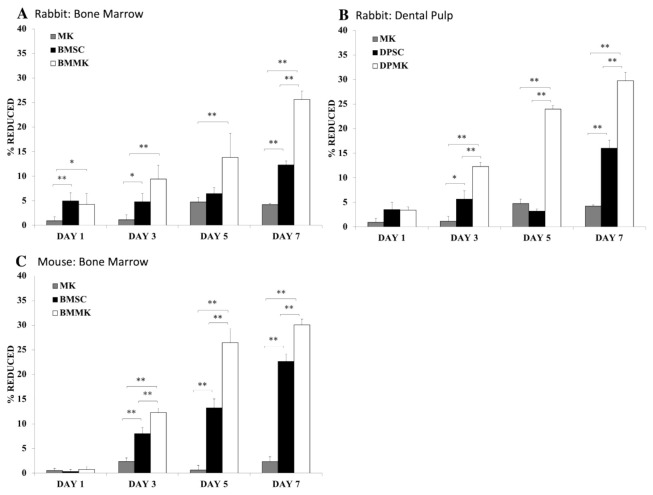
Shows MSC viability co-cultured with or without MKs. (**A**) shows rabbit MKs, rabbit BMSCs (BMSCs), and co-culture of rabbit MKs with rabbit BMSCs (BMMK). (**B**) shows rabbit MKs, rabbit DPSCs (DPSCs), and co-culture of rabbit MKs with rabbit DPSCs (DPMK). (**C**) shows mouse MKs, mouse BMSCs (BMSCs), and co-culture of mouse MKs with mouse BMSCs (BMMK). MSC viability was assessed on days 1, 3, 5, and 7. Increased levels of MSC viability are observed when MSCs are co-cultured with MKs at different time-points. Error bars reflect the standard deviation of the mean. A significant difference between the groups is indicated by the error bars: * = *p* < 0.05 and ** = *p* < 0.01 [125].

Using three-dimensional biodegradable hydrogel scaffolds has demonstrated a promising strategy [25,26,144,251]. Hydrogel scaffolds can provide a temporary structure for protection until BMSCs can differentiate and produce their own ECM [25,26,49,144]. Another strategy is a combined administration of BMSCs with growth factors or with other cell types. For instance, adding BMP-2 to BMSCs culture enhances survival rate and induces BMSC differentiation. A higher BMSCs survival rate is observed when combined administration of BMP-2 with immortalized mouse BMSCs are encapsulated in three-dimensional polymeric scaffolds, Figure 4 [47]. Another approach to increase BMSCs viability is demonstrated through co-culture of BMSCs with MKs, Figure 5A [125]. Maintaining BMSC high survival rate and potency after transplantation could increase their efficacy in vivo, therefore increasing new bone formation [220].

### 4.2. Dental Pulp Mesenchymal Stromal Cells (DPSCs)

Oral cavity is a rich source of MSCs with osteogenic potential. Craniofacial stem cells can be harvested from dental pulp, dental follicle, dental apical papilla, periodontal ligament, and gingiva [48,52,230,231,234,252,253,254,255]. Particularly, DPSCs are harvested from dental pulp tissue and used as stem cells for cranial bone regeneration. Scientists describe DPSCs as ectomesenchyme to distance DPSCs from BMSCs [54,57,256]. During embryogenesis, DPSCs originate from ectodermal cells that grow at periphery of neural tube and develop to express mesenchymal phenotype [50,52,53,236]. DPSCs can differentiate into multilineages, such as odontogenic, adipogenic, and neurogenic cells [234,237,257,258,259].

Similar to BMSCs, several bone markers are upregulated when DPSCs are differentiated into osteoblasts. Collagen type I, collagen type III, alkaline phosphatase, and osteocalcin are among these markers [48,260]. In contrast, proliferation rate and differentiation capacity can be distinguished between two MSC types [54,125,260,261,262,263]. Studies have demonstrated that DPSCs possess a higher metabolic and proliferative capacity than BMSCs [26,55,56,222,264]. A recent co-culture study of DPSCs with MKs shows higher levels of DPSC viability than BMSCs treated under the same condition at day 5, Figure 5B [125]. Similarly, Alge et al. studied differences and similarities between BMSCs and DPSCs harvested from rat animal model [54]. The authors concluded that DPSCs have a higher proliferation rate and higher expression of Alkaline Phosphatase (ALP) activity and calcium deposition than BMSCs [54]. Other research groups have reached similar conclusion [265,266]. Furthermore, DPSCs show no early senescence signs during in vitro expansion and passaging (replicative senescence) [51,222,267].

**Figure 6:**
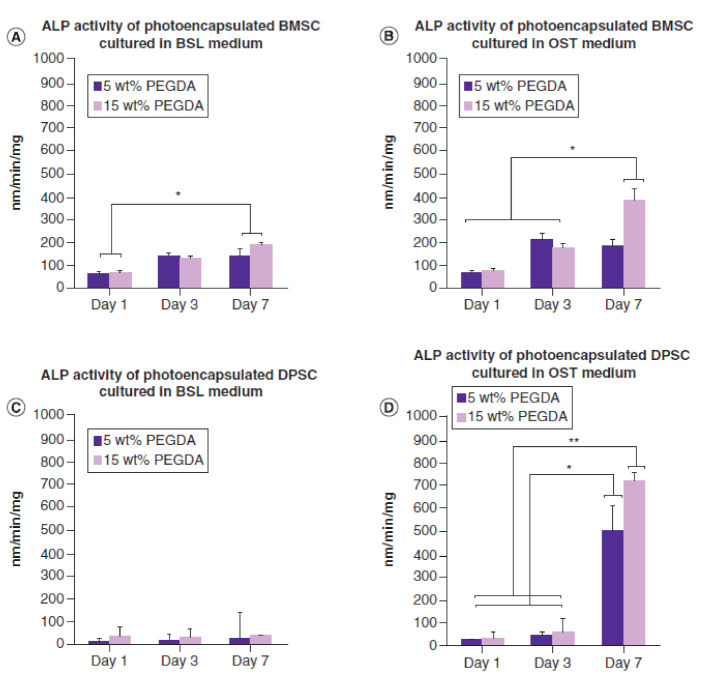
Shows ALP activity of photoencapsulated-MSCs (BMSCs and DPSCs) in 5 or 15 wt% PEGDA hydrogels, cultured in basal (BSL) or osteogenic (OST) medium. (**A**) Photoencapsulated-BMSCs in 5 or 15 wt% PEGDA hydrogels, cultured in BSL medium, show increased ALP activity levels at day 7 compared to day 1. (**B**) photoencapsulated-BMSCs in 15 wt% PEGDA hydrogels, cultured in OST medium, show increased ALP activity levels at day 7 compared to photoencapsulated-BMSCs in 5 or 15 wt% PEGDA hydrogels at days 1 and 3. (**C**) photoencapsulated-DPSCs in 5 or 15 wt% PEGDA hydrogels, cultured in BSL medium, show no difference in ALP activity at days 1, 3, and 7. (**D**) photoencapsulated-DPSCs in 5 or 15 wt% PEGDA hydrogels, cultured in OST medium, show increased ALP activity levels on day 7 compared to days 1 and 3. * and ** indicates that comparison values are significant (* *p <* 0.05 and ** *p* < 0.01). Error bars show ± SD [24].

In addition, DPSCs have been proposed as suitable MSCs for cranial reconstruction [268,269], perhaps due to their embryonic origin of craniofacial skeleton [54,57,231]. Investigators have shown that DPSCs enhance cranial bone regeneration in vivo when a cranial defect is created in various animal models [3,13,268,270,271,272]. In a previous study, photoencapsulated-DPSCs in thiol acrylate hydrogels show increased levels of ALP activity compared to photoencapsulated-BMSCs at day 7 [24], Figure 6. In addition, the study shows photoencapsulated-DPSCs in fast degrading thiol acrylate hydrogels demonstrate higher capacity inducing new bone formation in rabbit calvarial defects comparing to positive and negative control groups, 6 weeks post-implantation [24], Figure 3. The results of similar studies [3,13,24,268,270,271,272] using DPSCs to reconstruct cranial defects have demonstrated that DPSCs can be a reliable source enhancing cranial bone regeneration.

## 5. Protein-Based Therapy: Current Approaches and Potential Therapy

Protein therapy is a key strategy that several research groups currently explore to enhance craniofacial bone regeneration. Utilizing protein therapy in bone tissue engineering has showed promising and satisfactory results [251,273,274,275,276,277,278,279]. However, some patients experience complications and poor outcomes post-surgery [98,99,100,101,102,103,280]. Table 2 summarizes the advantages and disadvantages of current and potential proteins used for craniofacial bone tissue engineering.

### 5.1. Bone Morphogenetic Protein-Based Therapy

Bone Morphogenetic Proteins (BMPs) are multifunctional growth factors that belong to Transforming Growth Factors (TGF-β) superfamily [299,300,301]. BMPs are growth factors that regulate cellular functions and embryonic development of musculoskeletal tissues, including craniofacial development [282,302]. BMP-2 expresses during facial ectomesenchyme and tooth developments, as well as during early skull development [282,303].

Several types of BMPs are used to heal large bone defects: notably, BMP-2, BMP-6, and BMP-7. BMP-2 is considered a gold standard protein that is frequently used to regenerate critical-size bone defects. Since US Food and Drug Administration (FDA) has approved recombinant-human Bone Morphogenetic Protein-2 (BMP-2) [156,300,304], research studies have shown enhanced bone tissue regeneration in multiple animal models [65,274,275,276,299,300,305,306]. Culturing MSCs with BMP-2 has showed increased levels in bone markers, indicating that MSCs are differentiating into osteoblasts [251,273,274,283].

While BMP-2 shows desired outcomes in regenerating bone defects, higher risks in radiculitis, ectopic bone formation, osteolysis, and inferior global products are reported by a peer-review on 13 industry-sponsored BMP-2 projects [284]. Additionally, other reports have demonstrated controversial results of BMP-2 safety for in vivo applications. For instance, BMP-2 has been connected to higher risk developing cancer [98,99,100,101,102,103].

Furthermore, an ideal delivery of BMP-2 into defect site is a challenge to overcome in clinical applications. One reason may occur due to short half-life of BMP-2, usually 1 to 4 h [256,281]. In addition, robust release of BMP-2 post-implantation remain a challenge. However, effective methods and new approaches have been proposed and investigated to deliver BMP-2 effectively [281,307,308,309,310].

Nevertheless, better results are obtained when BMSCs are delivered along with BMP-2 [251,273,274,283]. Another challenge is that BMP-2 has been shown to boost growth of surrounding bone tissues, such as cartilage and tendon [311,312,313,314]. As a result, control release of BMP-2 is desired to eliminate untargeted tissue growth that may interrupt bone regeneration and cause cosmetic deformities.

**Figure 7:**
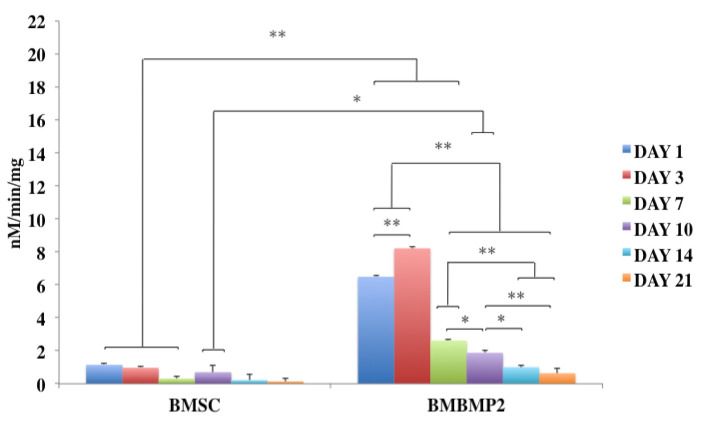
Shows ALP activity, bone mineralization marker, of immortalized mouse BMSCs photoencapsulated in visible light-cured thiol-acrylate hydrogels at days 1, 3, 7, 10, 14, and 21. *Y*-axis shows nano-molar/min/mg, *X*-axis shows control groups of photoencapsulated-BMSCs without BMP-2 (BMSCs) and experimental groups of photoencapsulated-BMSCs with BMP-2 (BMBMP2), (N = 4 ± SD). Statistical significance is set at: * = *p* < 0.05 and ** = *p* < 0.01 [47].

**Figure 8:**
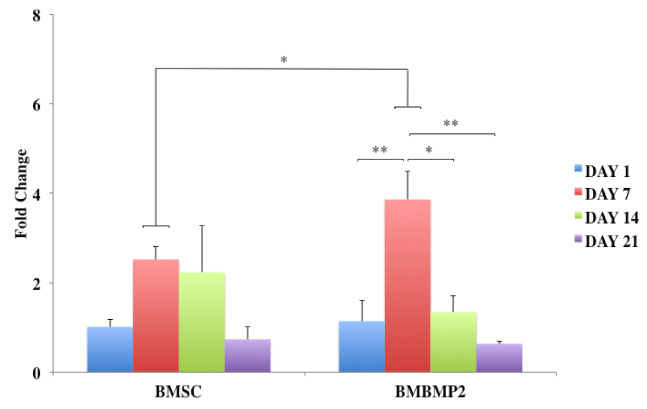
Shows *cFOS* expression, gene associated with cell cycle and growth, at days 1, 7, 14, and 21. *X*-axis shows control groups of photoencapsulated-BMSCs without BMP-2 (BMSCs) and experimental groups of photoencapsulated-BMSCs with BMP-2 (BMBMP2). *Y*-axis shows fold change. *cFOS* is expressed at day 7 in experimental group (BMBMP2) compared to control group (BMSCs). Statistical significance is set at: * = *p* < 0.05 and ** = *p* < 0.01, (N = 4 ± SD)—[47].

Despite challenges, BMP-2 has been a desired choice to regenerate large-size cranial defects. Studies have shown encouraging outcomes of BMP-2 in regulating human cranial osteoblasts by inducing MSC differentiation [315]. For instance, we have recently studied delivering immortalized mouse BMSCs using photoencapsulation method, with or without BMP-2, for craniofacial bone engineering applications [47]. Although negative control groups (photoencapsulated-BMSCs without BMP-2 (BMSCs)) and experimental groups (photoencapsulated-BMSCs with BMP-2 (BMBMP2)) are cultured in basal medium, an increased level of ALP activity is observed in experimental group at day 7 [47], Figure 7. Furthermore, expression of *c-Fos*, associated with cell cycle and growth [316], and confocal microscopy images show elevated viability levels of BMBMP2 compared to control group (BMSCs) [47], Figure 3 and Figure 8, respectively. The study shows ability of BMP-2, not only improving differentiation capacity of immortalized mouse BMSCs, but also enhancing viability.

Furthermore, a recent study shows BMP-2 can be a chemokine recruiting BMSCs in vitro and in vivo. Liu et al. show that BMP-2 stimulates migration of BMSCs by activating migration-related signaling pathways (CDC42/PAK1/LIMK1) in vitro [267]. Similarly, BMP-2 loaded on collagen sponge shows recruitment of BMSCs injected into circulatory system in vivo [267]. Using CDC42, an inhibitory silencing for migration-related signaling pathway, displays a significant decrease in BMSC recruitment [267]. Therefore, study shows ability of BMP-2 to recruit BMSCs and provides further understanding of BMP-2 benefits in vitro and in vivo.

Other related growth factors have been investigated as a potential candidate for cranial bone repair. For instance, Platelet-Derived Growth Factor (PDGF) and Fibroblast Growth Factor (FGF) have been explored in several studies [317,318,319,320]. Studies show increased levels in new tissue formation when Vascular Endothelial Growth Factor (VEGF) is combined with BMPs, due to a short half-life time of VEGF [321,322]. The authors concluded that combined growth factors stimulate osteoprogenitor cell differentiation and enhanced angiogenesis and regeneration of bone fractures [318].

### 5.2. Platelet-Rich Plasma (PRP)

Platelet-Rich Plasma (PRP) has been investigated in cranial bone regeneration [323,324]. PRP contains multiple growth factors, for instance, Platelet-Derived Growth Factor (PDGF) [315,321], TGF-β [287,292], Fibroblast Growth Factor (FGF) [292,323], Platelet-Derived Angiogenesis Factor (PDAF) [287,292], Vascular Endothelial Growth Factor (VEGF) [315,321], and Insulin-Like Growth Factor (IGF) [315,321]. PRP can be used alone, applied on a collagen sponge for sustained release [325] or encapsulated in hydrogel scaffolds, such as fibrin for craniofacial bone regeneration applications [305,306]. One of the advantages using PRP is to enhance cell migration and proliferation and angiogenesis [277]. In addition, preparing PRP as a hydrogel scaffold from patient reduces the risk of immune reaction and pathogen transmission from a donor [277,326].

PRP has some limitations in clinical applications. One of these limitations is that PRP, used as a natural gel, requires thrombin and calcium chloride to initiate the gelation in vitro. As a result, thrombin may have unsought effects by increasing the levels of two factors, V and XI, which can cause coagulopathies [291]. In addition, high concentrations of growth factors in PRP raise safety concerns [290]. For instance, adding multiple growth factors into defect sites at one time may increase potential risk of targeting native microenvironment of surrounding bone tissues [290]. Moreover, some studies have reported an inhibitory effect of PRP on osteoblasts [264,327]. Unlike BMP-2, PRP is not an osteoinductive factor [286]. Furthermore, there is a concern that PRP may cause infection during processing in vitro [286,289].

Despite challenges, PRP has proven to be an effective therapy in regenerating bone defects. Histological analysis by Xie et al. show a combined treatment of PRP, bone fragments, and BMSCs show a larger area of newly formed bone tissue compared to each component used alone [293]. Similarly, Oley et al. show higher lamellar bone growth when a large cranial bone defect is created in a rat animal model and scaffold with a hydroxyapatite combined with PRP is implanted [292]. These studies show efficacy of PRP as a potent growth factor regenerating bone defects.

## 6. Inducing MKs via Thrombopoietin: A Potential Therapy for Craniofacial Bone Defects

TPO is a protein that is produced by liver and kidney [301,328]. TPO is a primary growth and development factor that stimulates MK formation [329,330], Figure 2. TPO receptor, Mpl, has been identified in multiple cell lines, such as hematopoietic stem cells [107,108,109], megakaryocytic precursors [297], MKs [331], osteoclasts [120,122,125], osteoblasts [112,117,118,125], platelets [56,111,114], endothelial cells [304], hepatocyte progenitors [332], and cardiac cells [333]. Previously, TPO was used to treat thrombocytopenia, a low platelet count [330]. When TPO is engaged to Mpl receptor of HSCs, multiple intercellular signaling networks, such as PI3K/Akt/mTOR, MEK/MAPK, and JAK/STAT are activated. The activation course leads to mature and polyploid MKs that ultimately generate blood platelets [107,110,113,118,334,335,336]. These process takes approximately 5 days in humans and 2 to 3 days in rodents [335,337,338,339]. Recently, new evidence showed TPO receptor, Mpl, plays vital role in MK formation. The study shows that during steady-state hematopoiesis, TPO receptor (Mpl) rapidly simulates activation of HSC mitochondria to differentiate towards MK lineages [107].

While mechanisms have not yet been elucidated, growing evidence indicates that MKs play role in skeletal system, remodeling, and homeostasis [340]. For example, early research by Yan et al. shows that mice overexpressing TPO have an elevated MK level in bone marrow [341]. Increased MK count is associated with increased levels of bone formation [341]. Similarly, another study using animal model shows that mice with high MK count show an increased bone mass [112].

Moreover, studies have shown that MKs influence osteoblast and osteoclast proliferation and formation [108,112,113,114,115]. MKs have demonstrated a robust increase in osteoblast proliferation and bone formation [112,113]. A co-culture experiment of MKs with murine calvarial osteoblasts showed improvements in osteoblast proliferation by three- to six-fold, compared to control groups, osteoblasts cultured alone [114]. Other studies have demonstrated that MKs are vital in osteoclast formation [114,115,116,117,118,119]. In addition, osteoclasts are essential in bone remodeling and eliminate necrotic tissue in the early phases of bone repair [120,121]. An experiment investigating the effects of MKs on osteoclast formation shows prevention of osteoclast development in vitro [340]. Therefore, it is believed that MKs enhance bone mass by inhibiting bone resorption via decreasing osteoclastogenesis and increasing osteoblast proliferation, leading to a net increase in overall bone volume [112]. Furthermore, TPO has indirectly enhanced angiogenesis by increasing platelets (thrombocytes) and stimulating endothelial cell proliferation [342].

Although MSCs do not express TPO receptors (Mpl), MKs show a key role in regulating MSCs [125]. Recently, a study showed that prolonged co-culture of MKs with MSCs (BMSCs vs. DPSCs) enhanced MSC proliferation by two- to three-fold, compared to control groups, MSCs cultured alone [125]. However, the results also show MKs inhibit MSC differentiation into osteoblast lineage cells in vitro [125].

Successfully delivering and preserving high MSC count into defect site remains a challenge. Furthermore, immune system response and leakage of MSCs post-implantation led to lower prediction of MSC survival rate. Therefore, enhancing MSC viability post-implantation is desired. The evidence shows using TPO to induce MKs is a desired approach to investigate, to enhance regeneration of large cranial bone defects.

**Figure 9:**
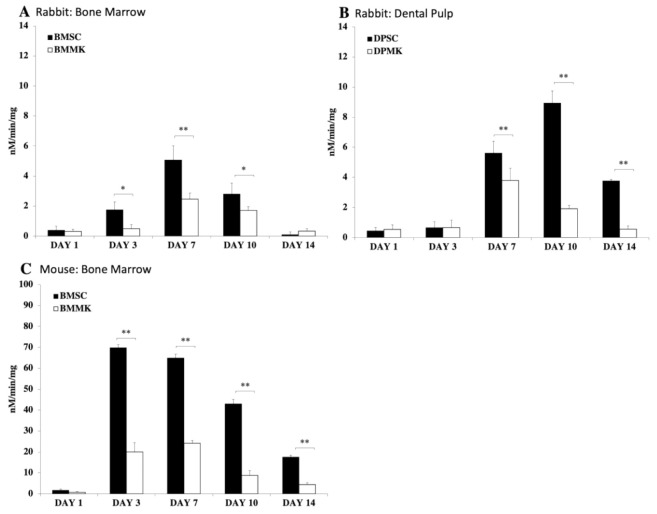
The ALP activity of MSCs co-cultured with or without MKs. (**A**) shows control group of rabbit BMSCs (BMSC) and experimental group of rabbit MKs co-culture with rabbit BMSCs (BMMK). (**B**) shows control group of rabbit DPSCs (DPSC) and experimental group of rabbit MKs co-cultured with rabbit DPSCs (DPMK). (**C**) shows control group of mouse BMSCs (BMSCs) and experimental group of mouse MKs co-cultured with mouse BMSCs (BMMK). ALP activity was assessed on days 1, 3, 7, 10, and 14. ALP activity was calculated per min and normalized per mg of proteins. Decreased levels of ALP activity are observed when MSCs co-cultured with MKs at different time-points. Error bars reflect standard deviation of the mean. Significant difference is indicated by error bars: * = *p* < 0.05 and ** = *p* < 0.01 [125].

**Figure 10:**
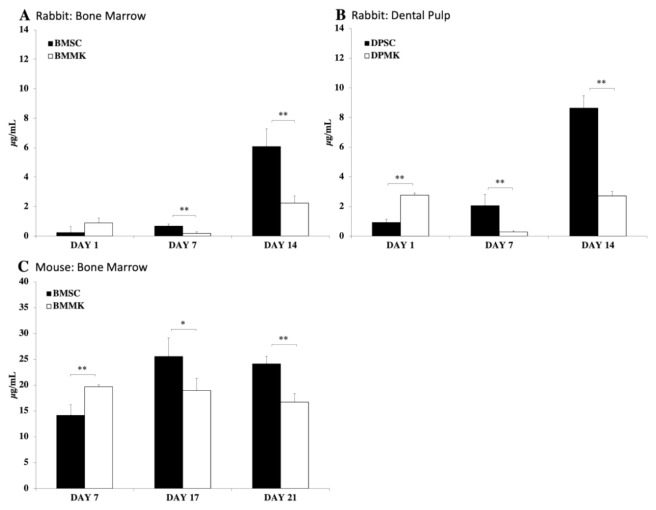
Calcium deposition of MSCs co-cultured with or without MKs. (**A**) shows control group of rabbit BMSCs (BMSCs) and experimental group of rabbit MKs co-culture with rabbit BMSCs (BMMK). (**B**) shows control group of rabbit DPSCs (DPSCs) and experimental group of rabbit MKs co-cultured with rabbit DPSCs (DPMK). (**C**) shows control group of mouse BMSCs (BMSCs) and experimental group of mouse MKs co-cultured with immortalized mouse BMSCs (BMMK). Figure 9A,B shows calcium deposition measured on days 1, 7, and 14, while Figure 9C shows calcium deposition measured on days 1, 17, and 21. Error bars reflect standard deviation of the mean. Significant difference between groups is indicated by error bars: * = *p* < 0.05 and ** = *p* < 0.01 [125].

Nevertheless, indirect impact of TPO may have a downside. For instance, directly injecting and delivering a high dose of TPO can increase platelet production [343]. Therefore, there is a potential risk of TPO to induce bone marrow fibrosis by increasing MK and platelet levels [122,126]. Another concern is that MKs have been shown to inhibit MSC differentiation [125]. Previously, we demonstrated prolonged co-culture of MKs with MSCs shows that MKs inhibit MSC differentiation into osteoblast lineages [125]. Furthermore, study reveals BMSCs, Figure 9A,C, and DPSCs, Figure 9B, co-cultured with MKs, have a significantly lower ALP activity expression than control groups, and similarly, a lower calcium deposition compared to control groups, Figure 10 [125]. Therefore, although MKs elevate BMSC and DPSC viabilities, MKs inhibit MSC differentiation into osteoblast linage cells [125].

TPO has demonstrated possibility increasing bone mass in animal models. Furthermore, several studies have shown co-culture of MKs increases osteoblast and MSC viabilities in vitro, although that MKs are relatively low, they account for approximately 0.01 to 0.05% of all nucleated bone marrow cells in humans [344,345]. The question is whether increasing MK count can enhance regeneration of large-size cranial defects in vivo. With evidence-based and promising practice, additional in vitro and in vivo studies are required to understand the effect of MKs on cranial bone regeneration.

## 7. Conclusions and Future Insights

Large cranial defects can result from a variety of conditions. Current approach to regenerate craniofacial bone defects is by pursuing tissue engineering approaches using bone graft substitutes combined with stem cells and growth factors. The uprising and rapidly developing field of stem cell technology and progress made in biomaterials science and technology have enabled cranial defect regeneration. Particularly, the use of growth factors, such as BMP-2 and PRP, have multiple advantages that activate MSCs to differentiate into osteoblasts lineage cells, though limitations exist. For instance, the need for a promising growth factor arises from excessive outcomes of BMP-2 and low survival rate of MSCs post-transplantation.

TPO is a megakaryocyte growth and platelet production. Studies have demonstrated that TPO may have a downside effect by inhibiting osteoclastogenesis and delaying MSC differentiation in vitro, as well as possibly inducing bone marrow fibrosis by increasing MK and platelet levels in vivo. However, co-culture studies of MKs show to enhance osteoblasts and MSC viabilities and maintain their phenotype in vitro, as well as increase bone mass in vivo.

Currently, there are multiple thrombopoietic agents (TPO-like agents) that FDA has approved to treat thrombocytopenia. Therefore, a large amount of information is known on the safety profiles of these agents. Although thrombopoietin to induce and increase MK count for craniofacial bone tissue engineering in humans requires FDA approval, it should not be expected to take as long as a newly tested protein for in vivo application. Therefore, inducing MKs using thrombopoietin for cranial bone regeneration may become a reality in the future.

## Figures and Tables

**Table 1:** Advantages and disadvantages of bone graft substitutes.

Bone Grafting	Descriptions	Advantages	Disadvantages	References
Autogenous Bone Grafting	Bone graft harvested from patient’s skeleton.	Highly biocompatible. Easy to integrate with surrounding tissues. Inducing osteogenesis, osteoinduction, and osteoconduction. Minimal potential risk of infection. Minimal potential risk of disease transmission. Available immediately for patients.	Requires two surgical procedures. Causes local pain at donor site. Extends patients’ recovery time. Requires long time to full recovery. Can leave a scar at harvested site. Patients may require taking medications for faster recovery. Low cost-effective.	[147,152,158,159,161,166,167]
Allogeneic Bone Grafting	Harvested from a donor of same species.	Ability to harvest similar bone tissue to lost bone tissue. Does not require a second surgery site. Eliminates donor site morbidity. Minimize requirements for patients to stay in the hospital for a long time to recover once donor match is found.	High risk of infection and transmitting viruses. Hard to find matching donor. Takes longer time to incorporate with the surrounding native tissue and prolonged healing. Losing mechanical strength during preparations, cleaning, and sterilization processes.	[134,155,166,168,169,170,171]
Xenogeneic Bone Grafting	Harvested from different species such as animals and plants.	Reduces potential risk of infection. Reduces potential risk of human disease transmission. No second surgery is required at the site. Unlimited supply.	Potential risk of transmitting diseases from different species such as animals and plants. Potential risk of undesired immune system reaction. Losing mechanical strength due to cleaning and sterilization processes. Poor outcomes have been reported.	[149,166,172,173]
Alloplastic Bone Substitutes	Synthetic biomaterials such as polymer, metal, ceramic or composite biomaterials.	Biocompatible, biodegradable, and bioresorbable materials. Minimizes donor sites morbidity and pain. Minimizes potential risk of disease transmissions. Ability to adjust mechanical properties according to desirable bone tissue, shape, and location. Unlimited supply of materials.	Requires longer time for defected bone to heal. Produces less bone volume. Lower osteoinduction and osteogenesis.	[160,161,164,166,170]

**Table 2:** Advantages and disadvantages of protein-based therapy.

Proteins	Descriptions	Advantages	Disadvantages	References
BMP-2	A multifunctional growth factor belongs to transforming growth factors (TGF-β) superfamily.	Induces MSCs to differentiate into osteoblasts. Ability to recruit BMSCs into the defect site. Promotes new bone formation. Induces osteoinductive factors during bone healing. Indirectly enhances angiogenesis.	Has a short half-life and challenge to control dose deliveries into the defect site. Higher risk of radiculitis, ectopic bone formation, and osteolysis. Generally poor global outcomes Contraventional regarding its safety. Expensive.	[251,256,267,273,274,281,282,283,284,285]
Platelet-rich Plasma	Derived from whole blood. Contains multiple potent growth factors.	Enhances cell migration and proliferation. Promotes angiogenesis. Efficacy in promoting new bone tissue. Reduces risk of virus transmission (autologous). Reduces immune system reactions (autologous).	Not an osteoinductive agent. Requires bovine thrombin and calcium chloride to initiate gelation in vitro. Contains several growth factors into the defect site. May affect surrounding and untargeted tissues. High risk of infection.	[264,277,286,287,288,289,290,291,292,293]
TPO	Produced by liver and kidney. TPO receptor (Mpl) has been identified on several cells such as HSCs, MKs, and platelets.	Induces HSCs to differentiate into mature MKs and ultimately into platelets. Increased bone mass via induction of MKs. Enhances osteoblast proliferation and increases bone formation. Enhances angiogenesis by increasing platelet production and stimulating endothelial cell proliferation. Produce high MK count that enhances MSCs proliferation and survival rate. Produce high MK count that maintains MSCs stemness.	Hard to control dose deliveries into defect location. Produces high MK count that inhibits MSC differentiation into osteoclast lineage cells. Induces bone marrow fibrosis by increasing MKs and platelets. Produces high MK count that contributes in inhibiting osteoclastogenesis.	[112,114,117,125,294,295,296,297,298]

## Data Availability

Figure 1 was generated from United States National Library of Medicine (PubMed) datasets (https://pubmed.ncbi.nlm.nih.gov/?term=Craniofacial+Bone+Tissue+Engineering+%2B+Mesenchymal+Stromal+Cells&sort=date), accessed on 26 September 2021.
